# Adaptive Immunity to *Cryptococcus neoformans* Infections

**DOI:** 10.3390/jof3040064

**Published:** 2017-11-21

**Authors:** Liliane Mukaremera, Kirsten Nielsen

**Affiliations:** Department of Microbiology and Immunology, Medical School, University of Minnesota, 689 23rd Ave SE, Minneapolis, MN 55455, USA; lmukarem@umn.edu

**Keywords:** *Cryptococcus*, adaptive immunity, dendritic cells, CD4, helper T cell type 1 (Th1), helper T cell type 2 (Th2), helper T cell type 17 (Th17), cytokines, immune reconstitution inflammatory syndrome (IRIS)

## Abstract

The *Cryptococcus neoformans*/*Cryptococcus gattii* species complex is a group of fungal pathogens with different phenotypic and genotypic diversity that cause disease in immunocompromised patients as well as in healthy individuals. The immune response resulting from the interaction between *Cryptococcus* and the host immune system is a key determinant of the disease outcome. The species *C. neoformans* causes the majority of human infections, and therefore almost all immunological studies focused on *C. neoformans* infections. Thus, this review presents current understanding on the role of adaptive immunity during *C. neoformans* infections both in humans and in animal models of disease.

## 1. Introduction

The *Cryptococcus* species complex is a group of related fungal pathogens that cause disease in both healthy and immunocompromised patients. A recent study proposed to separate the *Cryptococcus* species complex into seven species; *C. neoformans*, *C. deneoformans*, *C. gattii*, *C. bacillisporus*, *C. deutorogattii*, *C. tetragattii*, and *C. decagattii* [1]. The first two species (*C. neoformans* and *C. deneoformans*) were previously known as *C. neoformans* var *grubii* and *C. neoformans* var *neoformans* respectively, while the other five species (*C. gattii*, *C. bacillisporus*, *C. deutorogattii*, *C. tetragattii* and *C. decagattii*) represent what was previously known as *C. gattii* VGI to VGIV [1]. *C. neoformans* and *C. deneoformans* mainly cause infections in profoundly immunosuppressed patients such as individuals with advanced HIV-AIDS, various T cell defects, patients with chronic lung, renal and hepatic diseases, and patients receiving immunosuppressive therapy before organ transplantation [2,3,4]. However, the five *C. gattii* species can cause disease both in immunocompetent and immunocompromised individuals [5,6,7,8]. In *C. gattii* infections, approximately 60% of affected populations have underlying diseases including respiratory diseases, diabetes or hematological malignancy [9]. These differences in patient populations suggest that the *Cryptococcus* species may exhibit subtle differences in their interaction with the host. However, the vast majority of studies exploring immune responses in both humans and animal models have utilized *C. neoformans* infections. Thus, this review will primarily focus on *C. neoformans*. 

### 1.1. Dendritic Cells and Macrophages Connect the Innate and Adaptive Immune Systems during C. Neoformans Infection

*C. neoformans* is found in the environment throughout the world, and has been extracted from soil, bird droppings and decaying wood [10,11]. *C. neoformans* infection starts following inhalation of fungal spores. Upon entering the lungs, *Cryptococcus* cells are recognized by host innate immune cells such as dendritic cells (DCs), epithelial cells, endothelial cells, and alveolar macrophages. These DCs and macrophages ingest and destroy invading *Cryptococcus*, present *Cryptococcus* antigens to T cells, and produce mediators (cytokines and chemokines) that initiate and direct the adaptive immune response [12,13,14,15]. Depletion of resident pulmonary DCs and alveolar macrophages results in rapid deterioration and death of mice infected with *C. neoformans* [16]. Thus, alveolar macrophages and DCs play an important role in the initiation of anti-cryptococcal immune responses, and link the innate and adaptive immune system during *C. neoformans* infection. In addition to their role as a physical barrier, epithelial and endothelial cells also act as effector cells during *C. neoformans* infection. Epithelial cells produce cytokines in response to *C. neoformans* [17], while endothelial cells augment the anti-cryptococcal activity of polymorphonuclear leukocytes [18]. 

### 1.2. Dendritic Cells Are the Primary Antigen Presenting Cells during Cryptococcal Infection

The respiratory tract contains a dense network of DCs with antigen uptake and presentation as their primary function [19,20]. Mature DCs migrate to T-cell-rich areas of secondary lymphoid organs, and present antigens to naïve T cells [20,21]. In addition to presenting antigens to naïve T cells, DCs also produce cytokines that regulate the adaptive immune response [21]. The expression of major histocompatibility complex class II molecules on DCs is sufficient to stimulate naïve T cells [20], and mice lacking DCs fail to generate cytotoxic T lymphocyte responses to intracellular pathogens [22]. Depending on the type of co-stimulatory molecules expressed on DCs, they can induce differential helper T cell responses. Helper T lymphocytes (Th cells) that respond to fungal pathogens can be divided into three main groups; (a) helper T cell type 1 (Th1) that produce pro-inflammatory responses to kill intracellular pathogens, (b) helper T cell type 2 (Th2) associated with the promotion of antibody, eosinophilic, and anti-inflammatory immune responses, and (c) helper T cell type 17 (Th17) cells associated with mucosal immunity and autoimmune diseases [23,24,25]. Dendritic cells matured in the presence of IFNγ induce the formation of IL-12 producing Th1 cells, and IL-12 secreted by DCs promotes the formation of IFNγ-producing cells [26,27]. On the other hand, DCs expressing costimulatory molecules CD86 and OX40L induce the development of Th2 cells that produce IL-4, IL-5 and IL-13 cytokines [26,28,29]. This Th2 immune response leads to eosinophilic airway inflammation [30]. Confirming these observations, blocking CD86 decreased Th2 immune responses, demonstrated by low IL-4 and IL-5 cytokines as well as low airway eosinophilia [31]. The formation of IL-17-producing Th17 cells requires the costimulatory molecules CD28 and ICOS [32]. In the absence of IL-4 and IFNγ cytokines, IL-23 induce naïve cells to differentiate into Th17 cells, and the presence of IL-4 and IFNγ blocks this differentiation [32,33]. Th17 immunity has been associated with both protective and non-protective roles during fungal infections [34,35,36,37,38,39]. During *C. neoformans* infection, Th1 immune responses are beneficial and support pathogen clearance, whereas Th2 immunity enhances disease, and Th17 cells have been associated with both protection and increased disease, depending upon the model used [39,40,41,42,43,44,45,46,47,48]. 

In a mouse model of *C. neoformans* infection, lung DCs internalize cryptococcal cells within 2 h post-infection, and following this internalization, lung DCs express the maturation markers CD80, CD86 and major histocompatibility class II [14]. The stimulated DCs induce production of high levels of IL-2 cytokine in vitro when co-incubated with *C. neoformans* antigen-specific T cells compared to naïve T cells, demonstrating *C. neoformans* antigen presentation and T cell activation [14]. Myeloid DCs and Langerhans cells, but not lymphoid DCs, are the antigen presenting cells needed to induce a protective immune response during *C. neoformans* infection [49]. A subset of lung resident DCs, CD11^+^ conventional DCs, mediates the accumulation of pathological Th2 cells following pulmonary *C. neoformans* infection, and lymphoid priming is not required for pulmonary Th2 cell accumulation [47]. These data demonstrate that the type of antigen-presenting DCs is important in the polarization of Th-mediated adaptive immune responses. 

## 2. Cell-Mediated Immunity: T Cells

### 2.1. Importance of T Cells during Cryptococcal Infections

*C. neoformans* cause infections in immunocompromised patients, mainly individuals with HIV-AIDS. In non-HIV individuals, most patients who present with cryptococcosis have other underlying diseases or immunosuppression. These include patients receiving immunosuppressive medication before organ transplantation, patients on glucocorticosteroid therapy, patients with chronic hepatic, renal and lung diseases, and patients with idiopathic CD4 lymphocytopenia [3,4,50,51,52]. *C. neoformans* also infects patients suffering from X-linked hyper IgM syndrome with defects in their circulating T cells [53,54,55]. These observations clearly demonstrate that CD4 T cell-mediated immunity plays a critical role in controlling human cryptococcal infections. 

In vitro and animal studies complement observations from human infections, and allow further identification of the role of T cells in immunity against *C. neoformans*. Different T cell subsets are involved in the immune response to *C. neoformans* infection. Both CD4 and CD8 T cells can inhibit the growth of *C. neoformans* cells by either direct killing [56,57], or production of pro-inflammatory cytokines that recruit and activate other phagocytes to kill *C. neoformans* cells [58,59,60,61,62]. Regulatory T cells promote protection against *Cryptococcus* infection by suppressing detrimental Th2 immune responses [63,64,65]. Other T cell subsets such as Natural Killer (NK), Natural Killer T (NKT) and gamma delta T (γδ T) cells are also involved in the development of a protective immune response against *Cryptococcus* infection [66,67,68,69,70,71]. However, γδ T cells can also downregulate the protective Th1 immune response [72].

### 2.2. Cryptococcal Antigens Activate T Cell Maturation and Proliferation

Whole *C. neoformans* cells or cell extracts (membrane, cell walls and proteins) induce the proliferation of human naïve T cells [73,74]. Phagocytosis and protein processing by accessory cells are necessary for the presentation of *C. neoformans* antigen to T lymphocytes [75]. Previous studies demonstrated that both CD4 and CD8 T cells proliferate in response to various *C. neoformans* antigens [59,60], and that immunity to *C. neoformans* infection requires both CD4 and CD8 T cells [76]. In addition, both CD4 and CD8 T cells directly inhibit the growth of cryptococcal cells in vitro [77,78], and both types of cells can use granulysin to kill *C. neoformans* [56,57]. A number of studies found divergent observations in addressing whether CD8 T cell responses depend upon CD4 T cells. Studies in mice found that neither CD8 T cell expansion and recruitment, nor their ability to produce IFNγ cytokine required CD4 T cells [58,61]. However, the loss of CD4 T cells resulted in a hyperexpansion of CD8 T cells [59]. On the other hand, studies using human cells showed that (a) proliferation of CD8 T cells requires CD4 T cells [60], and (b) the ability of CD8 T cells to kill *C. neoformans* through granulysin requires CD4 T cells, accessory cells and IL-15 [56]. These conflicting observations suggest that either the activity of CD4 and CD8 T cells in response to *C. neoformans* is different between humans and mice, or that the recruitment/expansion and function of CD8 T cells are regulated differently with CD4 T cells regulating the ability of CD8 T cells to kill *C. neoformans* through granulysin, but having no effect on how CD8 T cells develop and are recruited. 

During cryptococcal infection, stimulated lung-infiltrating T lymphocytes secrete both Th1 (IFNγ, IL-2) and Th2 (IL-4, IL-5, IL-10) cytokines [61]. The absence of CD4 T cells is accompanied by the loss of T cells secreting IL-4, IL-5 and IL-10, but residual CD8 still produces IFNγ and IL-2 cytokines [61]. On the other hand, T cells from CD8 deficient mice produced similar IL-4, IL-5 and IL-10 levels as the control mice, but secreted lower levels of IFNγ [61]. Both CD4 and CD8 T cells produce IFNγ and TNFα cytokines in the lungs of infected mice [59]. These data suggest that both CD4 and CD8 T cells produce Th1 cytokines; however, CD4 T cells are the main or sole source of Th2 cytokines during *C. neoformans* infection. 

### 2.3. Regulatory T Cells (Tregs)

Tregs are a subset of CD4 T cells that suppress immune responses [79,80]. Tregs have been found to play both beneficial and harmful roles during most common fungal infections. In *Histoplasma* and *Candida albicans* infections, reduced Tregs were associated with increased pro-inflammatory cytokines that promoted fungal clearance [81,82]. However, another study found that Tregs enhance Th17 responses and clearance of *C. albicans* cells [83]. In addition, Tregs were found to suppress Th2 immune responses in the lungs of mice infected with *Pneumocystis* [84]. 

Similar to their role during *Pneumocystis* infection, recent studies showed that Treg cells suppress harmful Th2 immune responses during murine *C. neoformans* infection [47,63,64,65]. Treg-depleted mice showed increased mucus production, eosinophilia, IgE production, Th2 cytokines (IL-4, IL-5, IL-13), as well as increased fungal burden [63]. Confirming these observations, increasing Tregs during pulmonary cryptococcal infection resulted in reduced IgE production, decreased mucus production and Th2 cytokines [64]. The C-C chemokine receptor type 5 (CCR5) and IFN regulatory factor 4 (IRF4) are required for the localization of Tregs to infected lungs and subsequent suppression of Th2 effector cells [65]. 

### 2.4. Natural Killer T(NKT) Cells

NKT cells play an important role in inducing protective Th1 immune responses during *C. neoformans* infection. *C. neoformans* infection is followed by an accumulation of NKT cells in the lungs and MCP-1 chemokine contributes to this NKT cell induction, especially Vα14^+^ NKT cells [66]. The activation of NKT cells is thought to be through the presentation of cryptococcal lipid antigens by DCs [68]. NKT cells induce delayed type hypersensitivity after immunization with cryptococcal culture filtrate antigen [69], and play an important role in the development of protective Th1 immune responses following *C. neoformans* infection [66,67,68]. In addition, activation of Vα14+ NKT cells with α-galactosylceramide resulted in Th1 immune responses, shown by increased IFNγ production, and enhanced local resistance to *C. neoformans* infection [67]. 

### 2.5. Gamma Delta (γδ) T Cells

During pulmonary *C. neoformans* infection, γδ T cells accumulate in the lungs, a process that does not involve MCP-1 chemokine [72]. Deficiency in γδ T cells was followed by an increase in IFNγ production, suggesting that they downregulate protective Th1 immune responses [72]. In addition, the absence of both Th cells and CD8 T cells leads to γδ T cell overproduction associated with neutrophilia and enhanced disease [39]. However, in the absence of neutrophils, γδ T cells produce IL-17A cytokine associated with protective immune responses in mice immunized with an IFNγ-producing *C. neoformans* strain [71]. The discrepancies in the roles of γδ T cells during *C. neoformans* infection observed in the above studies [39,71,72] might be due to the use of different *C. neoformans* and mouse strains. 

### 2.6. Memory T Cells

Defects in CARD9 are accompanied by impaired accumulation of Natural Killer (NK) and memory T cells in the lungs and increased susceptibility to *C. neoformans* infection, suggesting that both NK and memory T cells contribute to the protective immune response against *C. neoformans* [70].

## 3. Antibody-Mediated Immunity against Cryptococcal Infections

The fact that both humans and rodents produce antibody responses reactive with cryptococcal proteins [85] suggests that antibody-mediated immunity might play an important role against *C. neoformans* infection. Naïve laboratory mice and rats have no serum antibodies reactive with cryptococcal proteins, but produce antibody responses after *C. neoformans* infection [85]. Interestingly, adult human sera contain antibodies to cryptococcal proteins and GXM regardless of the person’s HIV status, previous history of *C. neoformans* infection, or whether the sera came from individuals with or without potential exposure to *C. neoformans* [85,86]. In addition, by the age of 5, sera from immunocompetent children also contain antibodies reactive with various cryptococcal proteins [87], suggesting that exposure to *C. neoformans* and subsequent development of antibody responses occurs early in life. 

### 3.1. B Cells and Antibody-Mediated Immune Responses in Human Cryptococcal Infections

Previous studies reported conflicting observations about the role of antibody-mediated immune responses in controlling cryptococcal infections. Deficiency in B cells and antibody immune responses have been associated with a greater risk for developing cryptococcal infections both in HIV and non-HIV patients [5,88,89]. In HIV-patients, decrease in B cells expressing IgM is associated with cryptococcal infections [88]. In addition, lower IgG counts are associated with cryptococcal meningitis in non-HIV patients with normal T cell counts and ratios [5,89]. Cryptococcal meningitis also occurs in patients with X-linked hyper IgM syndrome, characterized by lower IgG, IgA and IgE [53,54,90,91,92]. X-linked hyper IgM syndrome is a genetic defect in the gene encoding CD40 ligands on activated CD4 T cells, and is required for normal B cells activation [90,91,93]. *C. neoformans* infections are also linked to a total absence of B cells, such as in Burton’s agammaglobulinaemia in non-HIV patients [94,95]. These examples show that B cells and antibodies play an important role in controlling cryptococcal infection. 

Yet, antibody responses can also enhance cryptococcal disease in humans. Autoantibodies, such as anti-IFNγ and anti-GM-CSF, are associated with infections in non-HIV patients with normal CD4 counts [96]. Specifically, anti-GM-CSF autoantibodies were associated with cryptococcal meningitis in one patient [96]. 

### 3.2. B Cells and Antibody-Mediated Immune Responses in Non-Human Cryptococcal Infections

Early mouse studies concluded that antibodies were not involved in protection against *C. neoformans* because B cell deficient mice had no differences in mortality or organ CFUs when compared to wild-type mice [97]. However, subsequent studies demonstrated that B cells and antibodies can play a role in either resistance or susceptibility to cryptococcal infection. Deficiency in B cells and IgM antibodies has been associated with increased lung fungal burden and enhanced susceptibility to *C. neoformans* infection [98,99,100]. In addition, IgG antibodies enhance the ability of murine splenic NK cells to kill *C. neoformans* [101]. Another study showed that although B cells are dispensable for the development of acquired resistance to *C. neoformans*, they play an important role in protection against systemic infection when T cell immunity is impaired [102]. Divergence in the observed role of B cells and antibodies during *C. neoformans* infection might be due to the use of different *C. neoformans* and mouse strains. For example, studies showing that B cells and antibodies have a protective role against *C. neoformans* used C57BL/6J, BALB/c and CBA/J mouse strains [98,99,100], while the study that showed that antibody responses are dispensable during *C. neoformans* infection uses Swiss albino mice [97], and the *C. neoformans* strains used in these studies were also different [97,98,99,100]. 

Antibodies can also act as opsonins to enhance phagocytosis of *Cryptococcus* cells [103,104,105,106,107,108,109,110]. Anti-β-glucan monoclonal antibody with the ability to bind *C. neoformans* cell wall inhibits cryptococcal growth and increases in vitro killing by human and murine peritoneal macrophages [111]. In addition, administration of this anti-β-glucan antibody to infected mice reduces fungal burden in the brain and liver [111], showing that passive administration of anti-*Cryptococcus* antibodies can protect against *C. neoformans* infection. The efficacy of passive administration of anti-*Cryptococcus* antibodies depends on the antibody dose and mouse strain. In BALB/c mice, the efficacy of passive antibody decreases with higher doses, while in CBA/J mice protection against *C. neoformans* infection is only observed at high antibody doses [100]. In addition to antibody dose and host genetics, the type of antibody also affects the ability to confer protection against *C. neoformans* infection. Antibodies such as IgG, IgM and IgA have been associated with protection against *C. neoformans* infection [100,101,103,108,109,110], while increased IgE is associated with a Th2 immune response that exacerbates disease [100,112,113,114]. The above observations show that antibody-mediated immunity against *Cryptococcus* infection is a complex process where protective or non-protective roles of antibodies against *C. neoformans* depend on the type of *C. neoformans* strain, host genetics, and immunoglobulin class.

## 4. Cytokine Responses during *C. neoformans* Infections

Cytokines are small secreted proteins that mediate the interaction and communication between different immune cell types. Different cell types can secrete the same cytokines and similar functions can be induced by different types of cytokines. In addition, a single cytokine can act on many cell types, thus deciphering the impact of a specific cytokine can be challenging. A wide range of cytokines and chemokines (cytokines with chemotactic activities) play a crucial role in protecting mice against cryptococcal infection. A common feature of these protective cytokines and chemokines is that they either induce the Th1 immune response, enhance the activity of other Th1-inducing cytokines, and/or suppressing Th2 immune responses (Table 1). Th2 cytokines mainly induce non-protective immune responses that enhance cryptococcal disease, whereas Th17 cells are associated with both protection and disease enhancement during *C. neoformans* infection (Table 1). 

### 4.1. Protective Cytokines

The main protective cytokines produced during cryptococcal infections include IFNγ, IL-12 and IL-2. These cytokines have been associated with protection both in humans and mouse models of *C. neoformans* infection. In mice, the absence or reduction of IFNγ, IL-12 and IL-2 correlated with increased lung and brain fungal burden, increased lung eosinophilia, reduced numbers of macrophages expressing inducible nitric oxide synthase, increased fungal dissemination to the brain and overall increased susceptibility to infection [40,42,44,115,116,117,118]. Treating mice with anti-CD40 and IL-2 increases protection from disseminated *C. neoformans* infection through IFNγ activation of microglial cells [119,120], and IL-2 activated T and NK cells directly inhibit the growth of *C. neoformans* in vitro [77]. In addition, the absence of IL-12 induces a switch from protective Th1 to non-protective Th2 cytokines [40,44]. Human studies validate the role of IFNγ, IL-12 and IL-2 cytokines in protecting the host against *Cryptococcus* infection. Recombinant IFNγ and IL-2 were used as adjunct therapy to successfully treat cryptococcal meningitis (CM) patients [43,121,122,123]. Similar to observations in mice, adjunct IFNγ therapy was associated with an improved rate of fungal clearance in the CSF of HIV-CM patients with a trend towards improved mycological and clinical outcome [43,122], and restoration of immunological parameters and a sustained clinical recovery [121]. The importance of IL-12 in human CM was shown by (a) a correlation between higher CSF levels of IL-12 cytokine and increased survival in AIDS patients with CM, and (b) an increased ability to produce IFNγ when human PBMCs were treated with IL-12 [124]. All three cytokines are known to play a major role in the induction of Th1 immune responses. IFNγ and IL-12 cytokines from innate immune cells stimulate the differentiation of helper T cells into Th1 cells, while IL-2 induces proliferation of T cells [125,126]. 

In addition to these three major protective cytokines, there is another group of cytokines that we classified as supportive cytokines because they induce or promote the three major protective cytokines (IFNγ, IL-12 and IL-2) or their protective role has been shown in either human or animal models of cryptococcosis, but not in both. These supportive cytokines include TNFα, IL-6, IL-8, IL-18, IL-23 and IP10. The ability to produce TNFα, IL-8, IL-6 and IP10 cytokines was associated with improved outcome in AIDS patients with CM [124,127,128,129,130,131]. In addition, IL-6 and IL-1β are the main cytokines involved in anti-cryptococcal resistance in the brain of infected mice [132]. IL-23 and IL-18 cytokines were shown to play a protective role in mice against cryptococcal infection [133,134,135]; however, their role in human infection has yet to be determined. In mice, the absence of IL-23 was followed by impaired recruitment of inflammatory cells and cytokine responses [133], while defects in IL-18 correlated with increased lung fungal burden and reduced IFNγ and IL-12 cytokines [134,135].

### 4.2. Non-Protective Cytokines

In general, Th2 cytokines such as IL-5 and IL-13 promote cryptococcal disease. In mice, both IL-5 and IL-13 are associated with increased lung fungal burden, pulmonary eosinophilia, and overall increased sensitivity to *C. neoformans* infection [42,47,112,115,136,140,149]. In addition, the absence of IL-13 correlated with an increase in production of IFNγ and IL-17, cytokines known to be protective against *C. neoformans* infection [112]. Confirming observations in mice, high IL-13 levels in the CSF are associated with increased mortality in HIV-infected patients with CM [141]. IL-5 has not yet been associated with human *C. neoformans* infection.

#### Cytokines Associated with IRIS

Immune reconstitution inflammatory syndrome (IRIS) is characterized by pathological excessive inflammatory responses that result from a rapid recovery of immune responses in HIV-CM patients after antiretroviral therapy (ART) initiation [150]. Two different types of cryptococcal IRIS have been recognized; “paradoxical” and “unmasking” IRIS. Paradoxical IRIS presents as a worsening of disease or recurrent disease despite microbiological evidence of effective antifungal treatment (negative cultures) [151,152,153]. Unmasking IRIS is characterized by the development of *Cryptococcus* disease after ART initiation emerging from previous asymptomatic sub-clinical infection during immune reconstitution [150,154,155]. Various cytokines have been associated with cryptococcal IRIS. The absence/reduction of serum pro-inflammatory cytokines such as TNFα, G-CSF, GM-CSF and VEGF (vascular-endothelial growth factor), and increase in serum IL-17 and IL-4, predispose AIDS patient with CM to developing subsequent CM-IRIS [138]. Increased risk of CM-IRIS is also associated with low CSF inflammation at the time of diagnosis [130]. However, at the time of CM-IRIS, there are significant increases in CSF levels of IFNγ, TNFα, G-CSF, VEGF and eotaxin compared to baseline levels within AIDS patients [130]. These observations demonstrate that the types of sample (serum vs. CSF) and the time of analysis (diagnosis, during or after treatment) play an important role in relating cytokine responses to either a protective or non-protective role during *Cryptococcus* infection. 

### 4.3. Cytokines/Chemokines with Varying/Conflicting Roles

Cytokines/chemokines, such as IL-4, IL-8, IL-10, IL-1β, MCP-1 (monocyte chemoattract protein 1), MIP-1α (macrophage inflammatory protein form 1 alpha) and RANTES (CCL5) have been associated with both protection and disease exacerbation during *Cryptococcus* infection.

#### 4.3.1. Cytokines/Chemokines with Beneficial Role in Mice, but Detrimental in Humans

Decreased levels of MCP-1 in the lungs of infected mice correlated with impaired macrophages and CD4 T cell recruitment, reduced TNFα and IL-6 production, and inhibition of fungal clearance [147], and early expression of MCP-1 is associated with protection against *C. neoformans* infection [136,148]. Similarly, mice defective in MIP-1α production showed eosinophilic pneumonia, high levels of Th2 cytokines (IL-4, IL-13), and a reduction in protective cytokines IFNγ and IL-12 [40,136,148]. In contrast, human studies show that high MIP-1α and MCP-1 are associated with less peripheral CD4, lower CSF lymphocytes number, high risk for developing IRIS and increased mortality within AIDS patients with CM [131,141]. Observations from these studies suggest that MCP-1 and MIP-1α play antagonistic roles in inducing a protective immune response against *C. neoformans* infection in humans and mice. An alternative explanation could be that these chemokines play different roles at different sites of infection. This alternative explanation is supported by the fact that cytokines and chemokines were measured in CSF and/or serum in humans [131,141], but in lung and brain tissues in infected mice [40,136,147,148].

#### 4.3.2. Cytokines with Contradictory Roles in Both Mouse and Human *C. neoformans* Infections

Three cytokines IL-4, IL-10 and IL-17 have been reported to be protective against *C. neoformans* infection, detrimental or not having any effect on the course of disease progression. Increased levels of IL-4 are associated with slower clearance of *C. neoformans* cells and increased death of infected mice [115,144], and IL-4 was absent in the brain of immune (protected) mice [136]. Similarly, high levels of serum IL-4 correlated with development of IRIS and subsequent death in AIDS-CM patients from Brazil and Uganda [124,138]. In contrast, high CSF IL-4 levels have been associated with a protective immune response in AIDS-CM patients from South Africa [131]. In addition to this, several other studies reported that IL-4 cytokine has no effect on *C. neoformans* disease whether in mice [42,117] or humans [145]. Similar observations have been made for IL-10. IL-10 has been associated with (a) enhanced disease in both mice [100,117,136] and humans [124,142], (b) protection against *C. neoformans* disease in humans [129,131], and (c) having no effect on *C. neoformans* disease in human patients [146]. Reduced IL-17 levels correlated with increased susceptibility in mice [112], and high IL-17 levels in the CSF of AIDS patients with CM correlated with better fungal clearance and improved clinical outcome [124,131]. In addition, IL-17 activates anticryptococcal macrophage functions [45]. In contrast, high serum IL-17 levels, in the absence of pro-inflammatory cytokines, predispose AIDS patients with CM to subsequent IRIS and death [138]. These observations suggest that for IL-4, IL-10 and IL-17, the type of host (human or mouse) and/or site of infection (lungs, blood, CSF) are important in determining whether these cytokines play a protective or non-protective role during *Cryptococcus* infection. In addition, the above human studies were done in different countries and continents, suggesting that different patient populations respond differently to *C. neoformans* infections. 

#### 4.3.3. Cytokines with Contradictory Roles in Only One System

IL-8 and RANTES have been associated with *Cryptococcus* disease only in human patients and infected mice respectively. High CSF IL-8 levels are associated with increased survival among AIDS patients with CM in several studies [124,128,129]; however, similar plasma IL-8 levels were observed between AIDS patients with CM and control individuals in another study [146]. Similarly, increased expression of RANTES in the brain is associated with protection against CM in mice [136], but high levels of RANTES in the lungs correlated with increased Th2 immune responses and enhanced disease in another study [47]. These observations again suggest a differential role of cytokines/chemokines at different sites during *Cryptococcus* infection.

## 5. Current Model of the Adaptive Immune Response to *Cryptococcus* Infection

**Figure 1:**
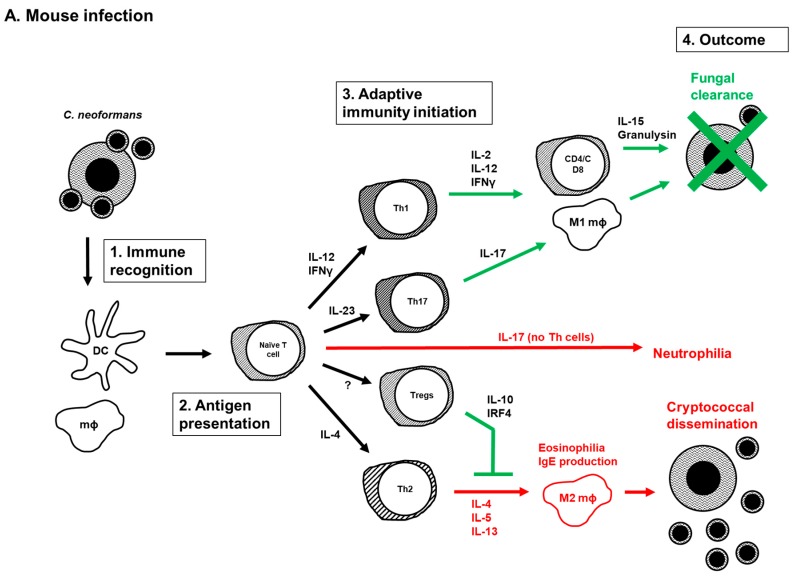

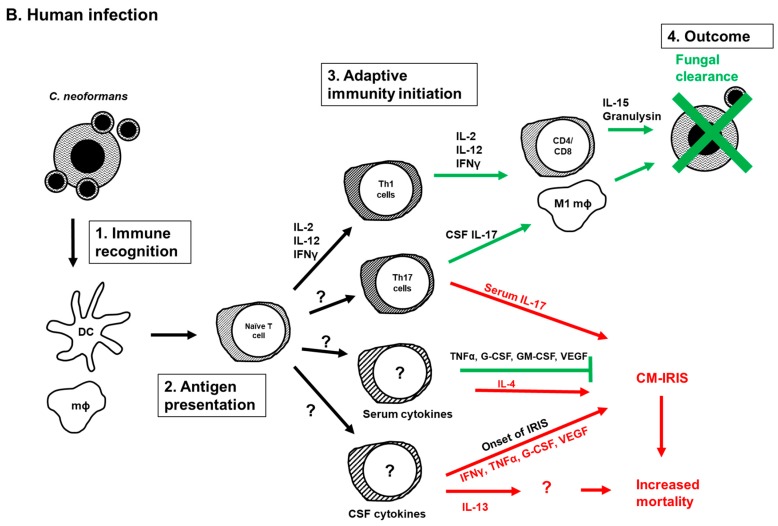
Adaptive immunity during *Cryptococcus* infection. Current understanding of initiation, development and function of adaptive immunity during *C. neoformans* infection in the mouse model (**A**) or in humans (**B**). (1) *C. neoformans* pathogen-associated molecule patterns (PAMPS) are recognized by innate immune cells (macrophages and DCs). This recognition triggers phagocytosis, antigen processing and presentation of *C. neoformans* antigens to naïve T cells by antigen presenting cells. (2) Antigen presentation induces activation and differentiation of naïve T cells into Th1, Th2, Tregs and Th17 cells. (3) The presence of IFNγ and IL-12 promotes Th1 differentiation, while the presence of IL-4, as well as expression of costimulatory molecules CD86 and OX40L induces Th2 differentiation. IL-23 induces Th17 differentiation in the absence of IFNγ and IL-4. (4) Th1 cells promote cryptococcal killing either by direct contact, or by producing the Th1 cytokines IFNγ, IL-12 and IL-2 that stimulate phagocyte recruitment and polarization to classically activated macrophages that eliminate *C. neoformans* cells. In contrast, Th2 immune responses mediated by IL-4, IL-5 and IL-13 result in increased eosinophilia and polarization of alternatively activated macrophages, and ultimately lead to the dissemination of *C. neoformans* cells and disease exacerbation. These Th2 immune responses can be blocked by the action of Tregs. In the absence of Th cells, IL-17 production intensifies cryptococcal disease through neutrophilia. Green arrows denote beneficial immune responses, whereas red arrows denote detrimental immune responses. CM (*Cryptococcus* meningitis), DCs (dendritic cells), IFNγ (interferon gamma), IL- (interleukin, IL-12: interleukin-12), IRF4 (IFN regulatory factor 4), IRIS (immune reconstitution inflammatory syndrome), Th (helper T cell), Tregs (regulatory T cells), Ø (macrophage), M1Ø (classically activated macrophage), M2Ø (alternatively activated macrophage), CD4 (helper T cell), CD8 (cytotoxic T cell), CD86 (costimulatory molecule 86), OX40L (costimulatory molecule OX40L, ligand of OX40 receptor on T cells).

A summary of our current understanding of the initiation, development, and function of adaptive immunity during *Cryptococcus* infection is presented in Figure 1. The model is based on information derived from *C. neoformans* experiments in mice (Figure 1A) as many aspects of the model are yet to be explored in humans (Figure 1B). Following infection, innate immune cells, mainly macrophages and DCs, recognize and phagocytose *C. neoformans* cells. Cryptococcal antigens are processed and presented to naïve T cells by antigen presenting cells (APCs). Naïve T cells will then differentiate into mature helper T cells. The type of cryptococcal antigens, co-stimulatory molecules on antigen presenting cells and presence of cytokines produced by innate cells determine whether naïve T cells differentiate into Th1, Th2 or Th17 cells. The presence of IFNγ and IL-12 induces the differentiation of naïve CD4 T cells into Th1 cells, while the presence of IL-4 and expression of costimulatory molecules CD86 and OX40L on APCs induce the formation of Th2 cells. In the absence of IFNγ and IL-4 cytokines, IL-23 induces the formation of Th17 cells, and these IL-17 producing T cells typically enhance protection in a healthy host. However, the Th17 cells exacerbate cryptococcal disease when both CD4 and CD8 T cells are lacking, such as in individuals with HIV/AIDS. IL-17 cytokine has also been associated with increased risk of developing CM-IRIS in HIV infected patients. Cytokines produced by Th1 and Th2 cells can in turn activate and enhance macrophage function. The presence of IFNγ induces the development of classically activated macrophages (M1), while IL-4 and IL-13 direct macrophage polarization into alternatively activated macrophages (M2) [112,114,137,156]. M1 macrophages are associated with protection against *C. neoformans* infection, whereas M2 macrophages enhance disease by increasing intracellular cryptococcal proliferation. In addition to Th cells, increased Tregs in the lungs of infected mice promote protection against *C. neoformans* infection by blocking detrimental Th2 immune responses [63,64,65].

## 6. Concluding Remarks

Current understanding of adaptive immunity against *Cryptococcus* mainly comes from studies that used mouse models of disease. The availability of a wide range of genetically defined (knockout and transgenic) mouse strains makes mice an invaluable tool to study different aspects of the interaction between the host immune system and the pathogen. However, observations in mice do not always translate to human infection. For example, Th2 immune responses correlate with increased *C. neoformans* disease in mice [47,112,136,140,157], but various studies in humans do not associate a Th2 immune response to enhanced disease [131,145,158]. One reason for this difference could be the different types of samples collected in humans (blood and CSF) and mice (predominantly lungs). In addition, we do not know the protective immune response in healthy humans because the only human studies to date focus on patients with *C. neoformans* infection.

Human studies focused on infection of the central nervous system because the majority of patients present to hospitals with CM. However, the recognition and immune responses to *C. neoformans* in human lungs is not known, although the lungs are the initial site of cryptococcal infection. Studies in mice show that change in *C. neoformans* morphologies such as titan cell formation in the lungs of infected mice affect *C. neoformans* virulence [159,160,161,162,163]. Specifically, increases in cell wall chitin content are associated with detrimental Th2 immune responses in the lungs that worsen cryptococcal disease [47]. It is not known whether changes in cell surface components affect human immune responses to *Cryptococcus*. Extensive work has been done in understanding the role and origin of various cytokines in anti-cryptococcal immunity in the mouse model of infection. However, the role and origin (immune cell subsets) of cytokines during human *Cryptococcus* infection are not well known. Although various cytokines have been associated with either improved patient survival or worsening of disease, their specific role in immunity against *Cryptococcus* is not well understood. It is also not known whether these cytokines are produced by innate or adaptive immune cells. Additionally, different subsets of immune cells play different roles during *Cryptococcus* infection in mice (Figure 1A). However, it is not known whether similar mechanisms occur in human patients. Further studies are needed to identify mechanisms underlying protective immune responses in humans and address unanswered questions.

It is important to note that the majority of immunological studies have focused on *C. neoformans* infections because they are the most prevalent. Yet recent studies suggest that *C. gattii* infections in both mouse models and humans do not behave the same as *C. neoformans* [143,164,165,166,167]. This is not surprising, based on the observation that *C. gattii* often causes disease in immunocompetent individuals. Thus, additional studies on immune responses during *C. gattii* infection are desperately needed to better understand the similarities and differences between the *Cryptococcus* species and how they cause diseases.

## Figures and Tables

**Table 1:** Schematic representation of cytokine function based on tissue analyzed.

		Human					Mice				References
Classification	Cytokines/Chemokines	Blood/Plasma/Serum			CSF		Lungs		Spleen	Brain	
Protective cytokines	IFNγ										[40,118,121,122,127,128,130,136]
	IL-12										[40,100,116,124]
	IL-2						NA		NA		[119,120,123]
Protection support cytokines	IL-6	NA					NA				[100,117,128,129,130]
	IL-18	NA			NA				NA		[134,135]
	IL-23	NA			NA		NA				[133]
	IP10								NA		[127,130,136,137]
	G-CSF								NA	NA	[71,128,130,138,139]
	GM-CSF						NA		NA	NA	[128,131,138]
	TNFα								NA		[41,121,128,130,136,138]
Non-protective cytokines	IL-5	NA			NA						[47,112,136,140]
	IL-13	NA								NA	[42,47,112,115,141]
Cytokines/ chemokines with varying roles	IL-1β						NA		NA		[136,142,143]
	IL-4										[42,112,115,117,124,131,136,138,144,145]
	IL-8						NA		NA	NA	[124,128,129,130,146]
	IL-10									NA	[47,100,117,124,127,129,131,142,146]
	IL-17									NA	[39,45,112,124,131,138]
	MCP-1	NA							NA		[131,136,147,148]
	MIP-1α	NA							NA	NA	[40,131,136,148]
	RANTES	NA			NA				NA		[47,136]

Blue (protective), yellow (neutral), red (non-protective), NA: not available.
